# Challenges and Opportunities for UAV-Based Digital Elevation Model Generation for Flood-Risk Management: A Case of Princeville, North Carolina

**DOI:** 10.3390/s18113843

**Published:** 2018-11-09

**Authors:** Leila Hashemi-Beni, Jeffery Jones, Gary Thompson, Curt Johnson, Asmamaw Gebrehiwot

**Affiliations:** 1 Geomatics Program, Department of Built Environment, North Carolina A&T State University, Greensboro, NC 27411, USA; jbjones1@aggies.ncat.edu (J.J.); aagebrehiwot@aggies.ncat.edu (A.G.); 2 North Carolina Emergency Management, Geodetic Survey; NC 27699-4298, USA; gary.thompson@ncdps.gov (G.T.); curt.johnson@ncdps.gov (C.J.)

**Keywords:** UAV, 3D flood mapping, remote sensing, digital elevation model, 3D modeling

## Abstract

Among the different types of natural disasters, floods are the most devastating, widespread, and frequent. Floods account for approximately 30% of the total loss caused by natural disasters. Accurate flood-risk mapping is critical in reducing such damages by correctly predicting the extent of a flood when coupled with rain and stage gage data, supporting emergency-response planning, developing land use plans and regulations with regard to the construction of structures and infrastructures, and providing damage assessment in both spatial and temporal measurements. The reliability and accuracy of such flood assessment maps is dependent on the quality of the digital elevation model (DEM) in flood conditions. This study investigates the quality of an Unmanned Aerial Vehicle (UAV)-based DEM for spatial flood assessment mapping and evaluating the extent of a flood event in Princeville, North Carolina during Hurricane Matthew. The challenges and problems of on-demand DEM production during a flooding event were discussed. An accuracy analysis was performed by comparing the water surface extracted from the UAV-derived DEM with the water surface/stage obtained using the nearby US Geologic Survey (USGS) stream gauge station and LiDAR data.

## 1. Introduction

Among the different types of natural disasters, floods are the most devastating, widespread, and frequent. Floods account for approximately 30% of the total loss caused by natural disasters [1]. For example, in October of 2016, Hurricane Matthew caused massive flooding in the eastern section of North Carolina, devastating towns such as Princeville, Lumberton, Smithfield, Kinston, Fayetteville, and Goldsboro. Many rivers in North Carolina surpassed record levels established by Hurricane Floyd in 1999. During the Hurricane Matthew flooding, 20 dams breached causing additional flooding in low-lying areas. In the City of Smithfield, the water treatment plant was completely inundated, leaving the residents without potable water. Accurate flood-risk mapping is critical in reducing such damages by correctly predicting the extent of a flood when coupled with rain and stage gage data, supporting emergency-response planning, developing land use plans and regulations with regard to the construction of structures and infrastructures, and providing damage assessment in both spatial and temporal measurements [2].

The technological development of Unmanned Aerial Vehicles (UAVs) has created a new tool for Geospatial data collection [3,4]. The advantage of UAVs, in comparison to traditional data acquisition approaches, is the ability to quickly deliver high spatial resolution imagery for a temporal event (e.g., the extent of flooding at a particular flood stage) [5]. UAVs are flexible and can be flown with different sensors that can be configured to detect a variety of potential data requirements, especially for areas with complex urban landscape as well as inaccessible areas due to hazardous environments. Additionally, UAVs can also be operated at a lower cost than manned aircraft for a localized photogrammetry project, and can even be used to revisit a site multiple times to track changes through an event, such as flooding. Thus, UAVs are cost-effective photogrammetry platforms that provide rapid deployment on-demand flood mapping for small project areas. The reliability and accuracy of such maps is dependent on the quality of the digital elevation model (DEM) and topography information [6,7,8]. High quality topography information and DEM are critical for flood-risk analyses for both hazard and vulnerability models [9]. Zazo et al. [10] show a small error in a DEM, especially in a flood-prone area, results in a significant change in the flood-risk map of that area. 

Many researchers have created and analyzed the quality of UAV-based DEM, DSM, and orthoimages for different applications [11,12,13,14,15,16]. UAV photogrammetry was investigated for topographic monitoring of coastal areas [17] and 3D mapping application [18,19]. The studies prove the advantage of UAV devices for the monitoring of morphological changes induced by coastal dynamics and urban changes. UAV-based photogrammetry when coupled with surveyed ground control and/or RTK Global Navigation Satellite System (GNSS)-based positioning can capture spatial data with a richness of detail [20] that can meet high accuracy American Society for Photogrammetry and Remote Sensing (ASPRS) standards, but not surveying standards. Although photogrammetry is a surveying and mapping method that has several advantages over conventional surveying (e.g., it provides a broad view of the project area and can be used in locations that are unsafe to access), it also has it disadvantages (e.g., it cannot map areas blocked by trees). Thus, photogrammetry has its applications where its level of accuracy would be sufficient, but could not replace those applications requiring a higher level of accuracy from conventional surveying. Şerban et al. [21] investigated the use of UAV technology along with Leica MultiStation GNSS surveying to generate a high-quality DEM of the major and minor riverbeds in the Someşul Mic basin to obtain certain hydraulic parameters to study flood-risk management practices. The quality of a UAV-based DEM for the characterizing and quantifying of some river habitat and morphology parameters has been studied by [22]. A 5-cm orthomosaic and a DEM with vertical RMSE of 8.8 cm in dry areas and 11.9 cm in submerged areas were generated. The DEM accuracy was sufficient to initialize and run a two-dimensional hydrodynamic model, River2D. The model results, including depth and velocity distributions, were combined with the mapped physical habitat features to estimate available habitat in terms of weighted usable area. Leitao et al. [8] demonstrated the applicability and the advantages of using UAVs to generate very high resolution DEMs for urban overland flow and flood modelling. They investigated the quality of DEMs for 14 UAV flights considering different flight altitudes, images overlapping, camera pitch, and weather conditions. The DEM quality (RMSE value) decreases as the flying height increases, however, this difference in DEM quality is relatively small [8,23]. The DEMs were generated with vertical accuracy of 0.1 to 0.2 m, which was about two to three times the UAV imagery ground sample distance (GSD). The research compared the best-quality UAV DEM to a conventional LiDAR-based DEM. The minimum, maximum, mean, and standard deviation of the elevation differences between the two DEMs were −0.468, 0.306, 0.06, and 0.119 m, respectively. Ajayi et al. [24] studied the accuracy of GNSS receivers mounted on a UAV platform for the generation of DEMs. The average RSME between the DEM coordinates and the GNSS coordinates was 0.0270 meters. Coveney et al. [2] sought to assess the accuracy range of UAV DEM and orthoimages based on the number of ground control points (GCPs). The research work showed that increasing the number of GCPs beyond one every two hectares has no improvement in accuracy or any other additional benefits. Thus, for those photogrammetrists who had been using GCPs at a higher density than one GCP for every two hectares can reduce their time installing and surveying GCPs as well as reduce their image processing time. Conversely, for those photogrammetrists who had been using GCPs at a lower density than one GCP per every two hectares should consider increasing their GCP density for increased accuracy, but this will mean more time spent on installing and surveying GCPs as well as increase image processing time. Despite the wide range of research work and methods available for the production of successive high quality DEMs and DSMs from UAV data, there are still some questions (with open answers) when dealing with UAV-based measurements [25]. This paper discusses on-demand DEM production during a flooding event and investigates the quality of the models by comparison with the water surface derived from USGS stream stage and the challenges of operating in low-quality photogrammetry conditions. Accurate monitoring and mapping of the DEM and flood extent are critical to assess flooding risk, develop comprehensive relief efforts immediately after flooding, and provide damage assessment in both spatial and temporal measurements. 

## 2. Study Area and Data

The Town of Princeville, a flood-prone area, is located along the Tar River in Edgecombe County in North Carolina. The study area was selected because:

(a) A UAV dataset was available from an inundated area of the town after Hurricane Matthew in 2016
(b) LiDAR data, the flood gauge information, as well as data about discharge in an upstream station were available in the area (these were used to assess and validate the result DEM)

Many parts of the town of Princeville are in the 1% annual-chance flood floodplain, which is commonly referred to by the misleading “100-year floodplain” term, and has historically been susceptible to floods. Between 1800 and 1958, the Tar River flooded this area seven times. In 1965, the Army Corps of Engineers built the levee along the stretch of the river from the northwest to the north border of the town. The flooding that occurred in October 2016 was not caused by the levee being breached, but rather by flood waters pouring in from areas not blocked by the levee.

**Figure 1:**
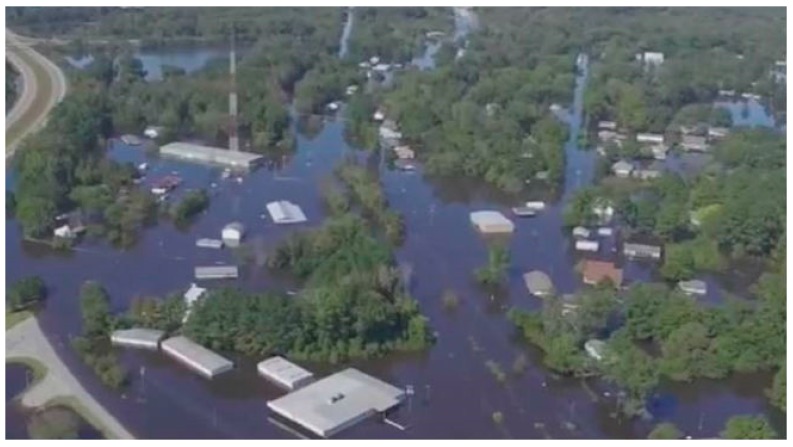
Study area: Princeville (North Carolina, USA) during Hurricane Matthew.

**Figure 2:**
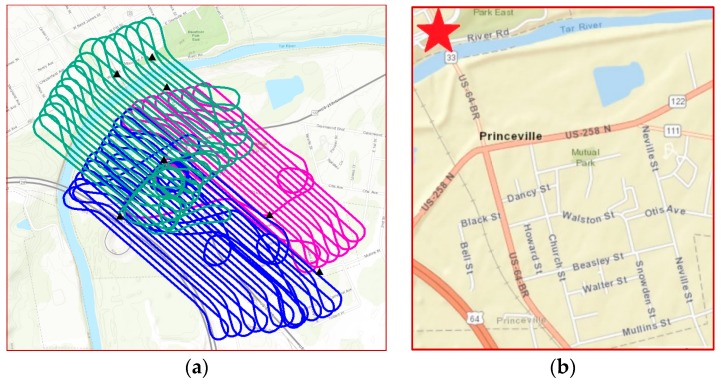
(**a**) Three flight blocks and distribution of the seven (7) ground control points (GCPs) in the study area, (**b**) Location of the 33661 USGS Gauge Station 02083500 TAR RIVER is shown by a red star (TARBORO vicinity map).

In October 2016, North Carolina Emergency Management (NCEM) overflew the northwest section of the Town of Princeville in three flight blocks on two different days (on 15 and 17 October) and at different times on each day to capture aerial imagery of areas flooded by Hurricane Matthew (Figure 1). Figure 2a shows the three flight blocks and distribution of GCPs in the study area. During the three flights (flight #1: 32 min, flight #2: 19 min, and flight #3: 27 min), 1,962 images were taken covering an area of 1.519 km^2^ with a 2.6 cm (1 inch) ground sampling distance (GSD). Aerial photographs were acquired at 80% forward overlap and 80% side overlap specifications. The flights were performed using a Trimble UX5 fixed-wing aircraft with imagery collected by a Sony a5100 camera outfitted with a 15 mm Voigtlander fixed focal length lens. Flying was conducted at 100 m above ground level at a cruising speed of 80 kph. The remaining sections of the town were not flown due to: (a) Not being able to find a suitable takeoff and landing location with a high enough vantage point to be able to observe the flights; (b) Not being able to see the aircraft had the remote pilot remained stationed where the flights for the northwest section were conducted.

Seven photo-controlled points were surveyed, but only four of them were visible in sufficient redundant photographs to be used for georeferencing the UAV images. The photo-controlled points had a vertical accuracy of 4 cm and a horizontal accuracy of 2.3 cm.

To assess the quality of UAV-based DEM, the Light Detection And Ranging (LiDAR) data, as well as the flood record at the USGS Tar River at Tarboro gauge station were collected. Lidar are widely regarded as the most suitable source for creating 3D models and DEM for use in flood modeling and mapping. In this research, LiDAR data was used to assess the quality and accuracy of UAV-based DEM.

QL2 LiDAR with 2 pulses per square meter (pls/m^2^) with an accuracy of 9.25-cm RMSEz was used, which aligns with the ASPRS 10-cm vertical accuracy class. The stage elevation at the USGS Surface Water Gauge Station, 02083500 Tar River at Tarboro, NC was collected. As part of the National Water Information system, USGS Gauge Stations collect time-series data for stream level, streamflow, reservoir and lake levels, surface-water quality, and rainfall. The Tar River at the Tarboro, NC site is located along US HWY 64 Business, directly across the Tar River from Princeville (Figure 2b). The proximity to the study area makes this site ideal for measurements of stream level during the flood event. Data is recorded every 15 min and is available via the USGS website (https://waterdata.usgs.gov/nwis/uv/?site_no=02083500&agency_cd=USGS). The elevation selected for the comparison (42.9 feet NAVD88) was taken from stream levels corresponding to photo timestamps of flight 2 (on 15 October, 14:30 EDT).

## 3. Data Processing

**Figure 3:**
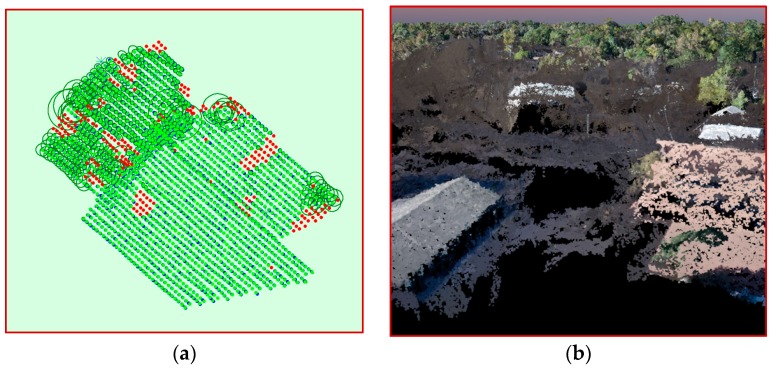
(**a**) Red points show the location of the images having calibration issues where selecting sufficient tie-points to reference the images was challenging due to the homogenous appearance of the water surface. (**b**) Initial point cloud includes considerable noise and artifacts.

Several methods such as structure-from-motion (SfM) and multiview-stereo have been developed to calibrate images in order to correct the geometrical deformations of the images, which are generally of poorer quality than photogrammetry metric camera used on traditional manned airborne platforms and then generate point clouds [26]. SfM methods require multiple overlapping images and use feature-based image matching methods for image-to-image registration and 3D surface construction [27]. High accuracy points cloud can be generated by using large image overlaps (80% to 90%) and matching multiple images in each point [28,29,30]. Data processing of the UAV-derived imagery was performed by Pix4Dmapper software. The camera used to capture the data was not a GNSS enabled camera, thus the raw images did not contain geolocation information and required georeferencing in addition to image calibration. The Trimble UX5 collected geotags for each image during the flights. The geotags were matched to the images and the three flight blocks were combined for georeferencing process due to the location of GCPs. Image calibration issues were found in large areas of open water where selecting sufficient tie-points to reference the images was challenging due to the homogenous appearance of the water surface. The uncalibrated images are shown as red points (Figure 3a).

There was considerable noise in the UAV-SFM point cloud (Figure 3b). The debris-filled floodwater appeared as shadowed areas, meaning there were considerable artifacts with elevations higher and lower than the actual water surface causing significant distortion when a DEM was generated. This issue, coupled with high vegetation, made it challenging to ascertain the water surface elevation. Furthermore, the automatic point cloud classification did not provide adequate feature resolution. The Pix4D point cloud classification is based on machine learning techniques requiring training on labeled data where both the geometry and the color information are used to assign the points of the densified point cloud in one of the predefined groups (e.g., building, ground, vegetation, etc.) [31].

**Figure 4:**
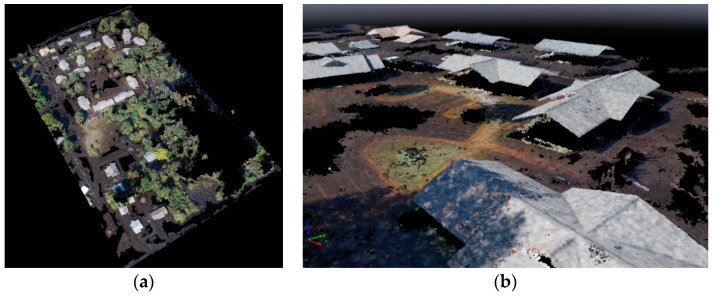
(**a**) Test area for post-processing and improving the quality of point cloud classification by removing the noises and water artifacts. (**b**) The point cloud after post processing.

To improve the DEM quality, and remove the water artifacts, a post-processing method was developed and performed. This method is based on a hydro flattening concept, assuming that the surfaces of water (lakes and, in our case, flooded areas) are flat. This method improved the water surface model by estimating a plane from the land/water interface in the point cloud, creating 3D breaklines, and a conflation methodology to remove water artifacts. In order to develop and implement the method and modify the point cloud classification as well as DEM, a smaller area was considered (Figure 4). This area included a variety of features such as visible rooftops, trees/high vegetation coverage, and open areas where the water/ground boundary was visible.

**Figure 5:**
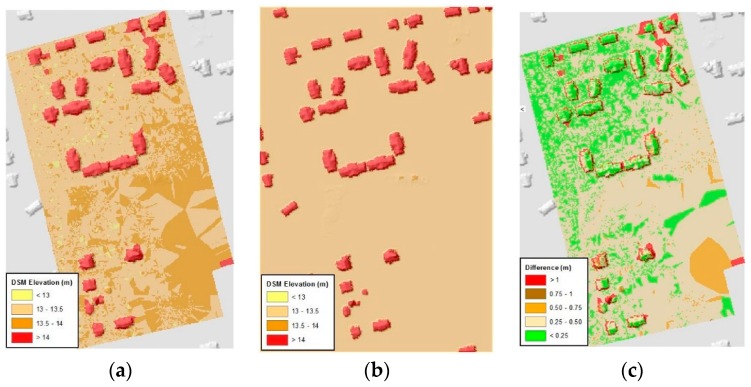
(**a**) UAV-based water surface raster (**b**) LiDAR-based water surface raster (**c**) comparison raster of UAV-based water surface to USGS Stream Level.

A DEM was generated for the study area. The most complete and uncontaminated features in the point cloud were structure roofs. The model was enhanced by the classification and noise removal method (Figure 5). To evaluate the suitability of the UAV-based water surface and validate the results, a comparison was made with a DEM, generated using the USGS Stream Level data and the LiDAR data. For this, a data driven approach [8] was implemented to synthesize a DEM and derive the water depths by intersecting a water mask of the observed flood event (USGS Stream Level data) with the LiDAR-based DEM generated before the flood event. This DEM was taken as a benchmark for the evaluation of quality of the UAV-based DEM results.

A qualitative quality assessment was done by a visual comparison between DEMs to identify problems such as discontinuities (Figure 5a,b). As expected, there are clear differences between the two DEMs in dense vegetated areas in the study area. Terrain estimation under dense vegetation was one of the most challenging issues in photogrammetry mapping, as the terrain was masked by the high vegetation, thus the software was unable to create sufficient terrain points in these areas.

For quantitative assessment, the elevation difference between the two DEMs was calculated on a pixel by pixel basis. For this, the UAV-based DEM that was originally computed at 3 cm (0.10 ft), was down-sampled at 90 cm using a bilinear interpolation method in order to match the resolution of LiDAR-based models. The elevation difference between the DEMs ($\Delta Z_{ij})$ was calculated by subtracting the UAV-based DEM ($Z_{UAV_{ij}}$) from the LiDAR-based DEM ($Z_{LiDAR_{ij}})$ at 90 cm resolution:

$$\;\Delta Z_{ij}=Z_{UAV_{ij}}-Z_{LiDAR_{ij}}\;$$

The results (Figure 5c) show that generally the UAV-based water surface was higher than the USGS Stream Level elevation. The mean difference was +27 cm (0.9 ft) with a standard deviation of ±15 cm (0.5 ft). As seen in Figure 5c, the surfaces were in better agreement in the northwest corner of the study area (<30 cm difference). This area is characterized by many structures; contrasted with the south and east areas where the study area was heavily wooded, where there was >30 cm difference between the surfaces.

## 4. Discussion

The on-demand deployment and small form factor, while being a significant advantage of UAV, also present considerable challenges. Environmental conditions during a flooding event, mainly the wind, pose the primary difficulty. Other challenges include low-quality photogrammetry conditions, camera calibration, point cloud classification, GCP availability, and processing time. Flight height, camera pitch, and image overlap are key factors affecting the results including point cloud and DEM. Photogrammetry best practices recommend that terrain mapping occur during the “leaf-off” seasons (late fall through early spring). However, flooding can occur at any time. Consequently, photogrammetrists must be prepared to handle imagery taken during “leaf on” conditions, which can affect the surface reconstruction in several ways. First, the terrain is masked by the high vegetation, thus the software is unable to create sufficient terrain points in these areas. Second, the high vegetation must be classified and removed from terrain analysis. Finally, with the high vegetation points removed, there are significant gaps in the point cloud, which make surface analysis challenging.

As mentioned earlier, SfM uses feature-based image matching for 3D reconstruction. Due to the homogenous appearance of the water surface, it was very difficult to process water bodies as there are insufficient tie-points to reference the images resulting in image calibration issues due to the homogenous appearance of the water surface. Moreover, the debris-filled floodwater appeared as shadowed areas causing significant distortion when a DEM was generated. 

Currently, the greatest challenge in using UAV-based photogrammetry for terrain modeling is point cloud classification. The automatic point cloud classification did not provide adequate feature resolution. Therefore, a post-processing classification method was required to approximate the water surface by creating 3D breaklines and a conflation methodology was required to remove water artifacts.

During the flood event, significant areas may be inaccessible and as a result unusable to place GCPs. Insufficient or poorly placed GCPs limit the ability of the indirect georeferencing to position the UAV-SFM point cloud within a spatial coordinate system. This then affects any calculations performed based upon the models derived from the point cloud. Improvement in direct georeferencing methods using dual frequency GNSS or multi-sensor system suggests that GCP-free UAV photogrammetry has great potential in the future [32,33,34,35]. Other complications of inaccessible areas are adequate area for take-off and landing of the UAV and pilot-in-command operating location.

Finally, many SfM applications are PC-based, which can limit the speed with which results are obtained, as opposed to parallel and cluster computation. The processing time depends on the image resolution, image content, overlap between images, chosen output resolution, and the computer used. For this project, it required approximately 33 hours to process the DSM, DTM, and Orthomosaic from the 1962 UAV images using, at 85% forward overlap and 80% side overlap, using Intel Xeon 3.7 Ghz processor, 16 GM RAM and NVIDIA Quadro GPU. Parallel and cluster computation can be an alternative for processing large amounts of the data.

Employing best practices and pre-planning can mitigate many of these challenges. A first step is to determine the purpose of the mission. By limiting the scope to one or two tasks, such as flood level determination in ungauged areas or aerial imagery based damage assessment, the spatial extent can be minimized and processing tasks prioritized in order to provide results in a timely manner.

Second, it is important to maximize the quality of the photogrammetry. If the required output is a 3D point cloud for flood level determination, seek open areas at the land/water interface that include structures. This will reduce the interference by high vegetation and large water surfaces and improve camera calibration. In addition, GCPs are required for indirect georeferencing methods and must be visible in multiple images; therefore, the flight lines must extend beyond the GCPs. The GCPs must be arranged to provide 3-axis orientation to the point cloud. As an alternative, direct georeferencing may reduce or eliminate the need for GCPs. Finally, efforts should be made to optimize the end and side overlap for the conditions and equipment used; too little overlap results in poorly calibrated images and too much overlap results in excess processing time. While these measures overcome many challenges, further development of point cloud classification algorithms, water surface classification in particular, and direct georeferencing will greatly improve the quality and speed of flood-assessment projects.

## 5. Conclusions

UAVs have been proven to be highly useful for mapping applications and have a great potential for fast and accurate on-demand DEM production in flood-assessment applications. However, there are failed image matching in low altitude image sets, because traditional processing methods are not flexible enough for UAV data. This issue, coupled with the inability of selecting accurate tie-points to reference the images in flooded areas, makes it challenging to create on-demand DEM during a flooding event. This research investigated the DEM production of UAV data captured after Hurricane Mathew in 2016 from a flood-prone area, the town of Princeville. An accuracy analysis was performed by comparing UAV-derived DEM with an integrated LiDAR and USGS stream level elevations. There is general agreement (less than 30 cm difference) between the models. More work is required for UAV-based DEM creation for flood applications due to the extremely challenging application requirements.

## References

[1] Adikari Y., Yoshitani J. Global Trends in Water-Related Disasters: An Insight for Policymakers. http://unesdoc.unesco.org/images/0018/001817/181793e.pdf.

[2] Coveney S., Roberts K. (2017). Lightweight UAV digital elevation models and orthoimagery for environmental applications: Data accuracy evaluation and potential for river flood risk modelling. Int. J. Remote Sens..

[3] Klemas V.V. (2015). Coastal and environmental remote sensing from unmanned aerial vehicles. J. Coast. Res..

[4] Lechner A.M., Fletcher A., Johansen K., Erskine P. Characterising upland swamps using object-based classification methods and hyper-spatial resolution imagery derived from an unmanned aerial vehicle. Proceedings of the XXII ISPRS Congress Annals of the Photogrammetry, Remote Sensing and Spatial Information Sciences.

[5] Popescu D., Ichim L., Caramihale T. Flood areas detection based on UAV surveillance system. Proceedings of the 19th International Conference on System Theory, Control and Computing (ICSTCC).

[6] Sanders B.F. (2007). Evaluation of on-line DEMs for flood inundation modeling. Adv. Water Resour..

[7] Kulkarni A.T., Mohanty J., Eldho T.I., Rao E.P., Mohan B.K. (2014). A web GIS based integrated flood assessment modeling tool for coastal urban watersheds. Comput. Geosci..

[8] Leitão J.P., Moy de Vitry M., Scheidegger A., Rieckermann J. (2016). Assessing the quality of digital elevation models obtained from mini unmanned aerial vehicles for overland flow modelling in urban areas. Hydrol. Earth Syst. Sci..

[9] Apel H., Aronica G.T., Kreibich H., Thieken A.H. (2009). Flood risk analyses—How detailed do we need to be?. Nat. Hazard..

[10] Zazo S., Molina J.L., Rodríguez-Gonzálvez P. (2015). Analysis of flood modeling through innovative geomatic methods. J. Hydrol..

[11] Uysal M., Toprak A.S., Polat N. (2015). DEM generation with UAV Photogrammetry and accuracy analysis in Sahitler hill. Measurement.

[12] Kršák B., Blišťan P., Pauliková A., Puškárová P., Kovanič Ľ., Palková J., Zelizňaková V. (2016). Use of low-cost UAV photogrammetry to analyze the accuracy of a digital elevation model in a case study. Measurement.

[13] Koci J., Jarihani B., Leon J.X., Sidle R. (2017). Assessment of UAV and Ground-Based Structure from Motion with Multi-View Stereo Photogrammetry in a Gullied Savanna Catchment. ISPRS Int. J. Geo-Inf..

[14] Rhee S., Kim T., Kim J., Kim M.C., Chang H.J. (2015). DSM generation and accuracy analysis from UAV images on river-side facilities. Korean J. Remote Sens..

[15] Rock G., Ries J.B., Udelhoven T. Sensitivity analysis of UAV-photogrammetry for creating digital elevation models (DEM). Proceedings of the Conference on Unmanned Aerial Vehicle in Geomatics.

[16] Ruiz J.J., Diaz-Mas L., Perez F., Viguria A. (2013). Evaluating the accuracy of DEM generation algorithms from UAV imagery. Int. Arch. Photogramm. Remote Sens. Spat. Inf. Sci..

[17] Gonçalves J., Henriques R. (2015). UAV photogrammetry for topographic monitoring of coastal areas. ISPRS J. Photogramm. Remote Sens..

[18] Nex F., Remondino F. (2014). UAV for 3D mapping applications: A review. Appl. Geomat..

[19] Küng O., Strecha C., Beyeler A., Zufferey J.C., Floreano D., Fua P., Gervaix F. The accuracy of automatic photogrammetric techniques on ultra-light UAV imagery. Proceedings of the Unmanned Aerial Vehicle in Geomatics.

[20] Barry P., Coakley R. (2013). Accuracy of UAV photogrammetry compared with network RTK GPS. Int. Arch. Photogramm. Remote Sens. Spat. Inf. Sci..

[21] Şerban G., Rus I., Vele D., Breţcan P., Alexe M., Petrea D. (2016). Flood-prone area delimitation using UAV technology, in the areas hard-to-reach for classic aircrafts: Case study in the north-east of Apuseni Mountains, Transylvania. Nat. Hazard..

[22] Tamminga A., Hugenholtz C., Eaton B., Lapointe M. (2015). Hyperspatial remote sensing of channel reach morphology and hydraulic fish habitat using an unmanned aerial vehicle (UAV): A first assessment in the context of river research and management. River Res. Appl..

[23] Udin W.S., Ahmad A. Assessment of photogrammetric mapping accuracy based on variation flying altitude using unmanned aerial vehicle. Proceedings of the IOP Conference Series: Earth and Environmental Science.

[24] Ajayi O.G., Salubi A.A., Angbas A.F., Odigure M.G. (2017). Generation of accurate digital elevation models from UAV acquired low percentage overlapping images. Int. J. Remote Sens..

[25] Colomina I., Molina P. (2014). Unmanned aerial systems for photogrammetry and remote sensing: A review. ISPRS J. Photogramm. Remote Sens..

[26] Zarco-Tejada P.J., Diaz-Varela R., Angileri V., Loudjani P. (2014). Tree height quantification using very high resolution imagery acquired from an unmanned aerial vehicle (UAV) and automatic 3D photo-reconstruction methods. Eur. J. Agron..

[27] Westoby M.J., Brasington J., Glasser N.F., Hambrey M.J., Reynolds J.M. (2012). Structure-from-Motion’ photogrammetry: A low-cost, effective tool for geoscience applications. Geomorphology.

[28] Baltsavias E., Gruen A., Eisenbeiss H., Zhang L., Waser L.T. (2008). High-quality image matching and automated generation of 3D tree models. Int. J. Remote Sens..

[29] Gülch E. Advanced Matching Techniques for High Precision Surface and Terrain Models. http://www.ifp.uni-stuttgart.de/publications/phowo09/300Guelch.pdf.

[30] Rosnell T., Honkavaara E. (2012). Point cloud generation from aerial image data acquired by a quadrocopter type micro unmanned aerial vehicle and a digital still camera. Sensors.

[31] Becker C., Häni N., Rosinskaya E., d’Angelo E., Strecha C. (2018). Classification of aerial photogrammetric 3D point clouds. Photogramm. Eng. Remote Sens..

[32] Elsharkawy A.S., Habib A.F. Error Analysis for the Airborne Direct Georeferincing Technique. Proceedings of the International Archives of the Photogrammetry, Remote Sensing and Spatial Information Sciences.

[33] Gabrlik P., Cour-Harbo A.L., Kalvodova P., Zalud L., Janata P. (2018). Calibration and accuracy assessment in a direct georeferencing system for UAS photogrammetry. Int. J. Remote Sens..

[34] Tulldahl H.M., Bissmarck F., Larsson H., Grönwall C., Tolt G. Accuracy evaluation of 3D lidar data from small UAV. Proceedings of the Electro-Optical Remote Sensing, Photonic Technologies, and Applications IX.

[35] Stöcker C., Nex F., Koeva M., Gerke M. Quality assessment of combined IMU/GNSS data for direct georeferencing in the context of UAV-based mapping. Proceedings of the International Conference on Unmanned Aerial Vehicles in Geomatics.

